# Pharmacogenomics: an opportunity for personalised psychotropic prescribing in adults with intellectual disabilities

**DOI:** 10.1192/bjo.2022.554

**Published:** 2022-08-17

**Authors:** Bhathika Perera, Charles Steward, Ken Courtenay, Timothy Andrews, Rohit Shankar

**Affiliations:** Barnet, Enfield and Haringey Mental Health NHS Trust, London, UK; Congenica Ltd, Cambridge, UK; Oxford Health NHS Foundation Trust, Oxford, UK; Peninsula School of Medicine, University of Plymouth, UK; and Cornwall Partnership NHS Foundation Trust, Truro, UK

**Keywords:** Intellectual disabilities, genetics, polypharmacy, psychotropics, discrimination

## Abstract

There is growing evidence for the use of pharmacogenomics in psychotropic prescribing. People with intellectual disabilities are disproportionately prescribed psychotropics and are at risk of polypharmacy. There is an urgent need for safeguards to prevent psychotropic overprescribing but it is equally crucial that this population is not left behind in such exciting initiatives. Understanding how genetic variations affect medications is a step towards personalised medicine. This may improve personalised prescribing for people with intellectual disabilities, especially given the high rate of psychiatric and behavioural problems in this population. Our editorial explores opportunities and challenges that pharmacogenomics offers for the challenges of polypharmacy and overprescribing of psychotropics in people with intellectual disabilities.

The Human Genome Project and derivative studies such as the UK 100 000 Genomes Project are providing researchers and clinicians with unprecedented insights into how advances in human genomics can lead to improved diagnosis and pharmacological interventions.^1^ This has led to the use of terms such as precision medicine or personalised medicine, which describe how recent advances in genomic medicine can help to improve effectiveness of drug therapy affected by individual genetic variants. Thus, advances in genomic medicine, supported by rigorous research methodologies, are leading to new discoveries on how pharmacological treatments can be used to treat diseases and modify disease trajectory.

## Pharmacogenomics and psychotropic medications

Psychotropic medications are widely used to treat various mental illnesses. Despite the strong evidence base for their effectiveness, they are often associated with multiple side-effects, rare life-threatening adverse events, drug interactions and variable treatment response from one individual to another. Understanding of the genetic influences on receptor activity, along with the pharmacokinetics and pharmacodynamics of psychotropic medications, has improved markedly over recent decades. Improved understanding of how metabolic pathways such as cytochrome P450 affect the metabolism of psychotropic medications and of the genes that encode them has led to a deeper understanding of the action of medications. Single nucleotide polymorphism (SNP) studies have enabled patient stratification as poor metabolisers, intermediate metabolisers, normal metabolisers, rapid metabolisers and ultra-rapid metabolisers.^2^ This is deemed important as poor metabolisers are considered to be at increased risk of developing toxicity, whereas ultra-rapid metabolisers may not achieve a therapeutic dose. Understanding of biotransformation enzyme variants has also added to the expanding knowledge, providing insight into how enzyme variants can expedite the metabolism of specific antidepressants and how ethnicity should be considered when prescribing.^3^

## Intellectual disabilities, mental health problems and psychotropics

Intellectual disabilities are a group of conditions that affect higher-order intellectual functioning beginning in childhood and leading to functional impairment. People with intellectual disabilities are at increased risk of comorbid psychiatric, neuropsychiatric and other neurodevelopmental disorders (Table 1). These may lead to complex behavioural and emotional clinical presentations that require challenging treatment decisions combining pharmacological and non-pharmacological strategies. Although there are higher levels of severe mental illness in populations with intellectual disabilities, psychotropic prescribing far exceeds the prevalence rates of mental illnesses for which such medication is indicated. This calls for better understanding of the prescribing of psychotropic medications, especially given the longer-term use in people with intellectual disabilities.

**Table 1:** Psychiatric, neurodevelopmental and neuropsychiatric comorbidity and their prevalence in people with intellectual disabilities

Comorbid disorder	Prevalence	Reference
Severe mental illness	10 times the general population level	Cooper SA et al. Multiple physical and mental health comorbidity in adults with intellectual disabilities: population-based cross-sectional analysis. *BMC Fam Pract* 2015; **16**: 110. Perera B et al. Mental and physical health conditions in people with intellectual disabilities: comparing local and national data. *Br J Learn Disabil* 2020; **48**: 19–27.
Any mental health condition	Odds ratio 7.1 (95% CI 6.8–7.3)	Hughes-McCormack LA et al. Prevalence of mental health conditions and relationship with general health in a whole-country population of people with intellectual disabilities compared with the general population. *BJPsych Open* 2017; **3**: 243–8.
Psychosis	2.6% (95% CI 1.8–3.8) to 4.4% (95% CI 3.2–5.8) depending on criteria used	Cooper SA et al. Psychosis and adults with intellectual disabilities: prevalence, incidence, and related factors. *Soc Psychiatry Psychiatr Epidemiol* 2007; **42**, 530–6.
Depression	17.0% (95% CI 16.8–17.2)	Branford D et al. Antidepressant prescribing for adult people with an intellectual disability living in England. *Br J Psychiatry* 2022; **221**: 488–93.
Anxiety	3.8% (95% CI 2.7–5.2)	Reid KA et al. Prevalence and associations of anxiety disorders in adults with intellectual disabilities. *J Intellect Disabil Res* 2011; **55**: 172–81.
Attention-deficit hyperactivity disorder	19.6%	La Malfa G et al. Detecting attention-deficit/hyperactivity disorder (ADHD) in adults with intellectual disability: the use of Conners’ Adult ADHD Rating Scales (CAARS). *Res Dev Disabil* 2008; **29**: 158–64.
Autism spectrum disorder	20–30% comorbidity	Emerson E et al. *The Estimated Prevalence of Autism among Adults with Learning Disabilities in England*. Improving Health and Lives Learning Disabilities Observatory, 2010.
Epilepsy	22.5%	Robertson J et al. Prevalence of epilepsy among people with intellectual disabilities: a systematic review. *Seizure*, 2015; **29**: 46–62. Shankar R et al. Epilepsy, an orphan disorder within the neurodevelopmental family. *J Neurol Neurosurg Psychiatry* 2020; **91**: 1245–7.

## Clinical validity

Pharmacogenomics is used in many ways. There is growing interest in evaluating a physician's ability to predict a person's response to drug therapy using DNA sequencing that could potentially aid in choosing the best pharmacological agents and doses in a more informed fashion for the person. Psychiatric genetics in general has generated some promising results on genetic variations associated with major psychiatric disorders and treatment outcomes. Despite these successes, psychiatry still lags behind other fields of medicine in translating existing knowledge into diagnostic genetic tests that could facilitate the early diagnosis and accurate classification of disorders.^4^ The validity of pharmacogenomic testing and its clinical utility in people with intellectual disabilities and mental disorders is also still at an early stage of development.

## Can pharmacogenomics help people with intellectual disabilities and mental disorders?

There is an ongoing debate on whether pharmacogenomic testing has the potential to make a difference in clinical practice. The Clinical Pharmacogenetics Implementation Consortium (CPIC) has US Food and Drug Administration (FDA) approved guidelines advising clinicians to consider genetic testing to ascertain metabolism profile for specific psychotropic medications. The 100,000 Genomes Project pilot investigators suggest that if similar measures were implemented in the UK, a person's diagnosis and treatment odyssey would be reduced from years to months.^1^ The authors believe integrating multiple data-sets such as the UK Clinical Practice Research Datalink (CPRD) (https://www.cprd.com) and the UK Biobank would allow researchers to correlate real-time prescribing practices with a person's genotype.

## What are the barriers to implementing pharmacogenomics in clinical practice for people with intellectual disabilities?

Evidence on the use of pharmacogenomic testing in people with intellectual disabilities is limited.^5^ There is a lack of evidence on whether pharmacogenomic knowledge will translate into tangible clinical differences in prescribing.^6^ There is also limited evidence on the modelling of benefits and costs associated with pharmacogenomics testing. As a result, pharmacogenomics is not included in treatment guidelines. Other challenges include the mental capacity of the individual to make an informed decision on the use of genetic testing. Furthermore, public perception of pharmacogenetic testing has not been tested yet.

## Whole exome sequencing (WES) and whole genome sequencing (WGS)

In recent years, the cost associated with pharmacogenomic testing has decreased greatly. Many pharmacogenomic samples can be collected using a minimally invasive buccal swab and processed within 7 days, allowing for real-time medication adjustments. However, such testing has typically been carried out by targeted methods such as quantitative polymerase chain reaction (qPCR) or SNP array and independently of both WES and WGS tests. WES is currently the most important method of genomic investigation used to identify causal genetic variants for the diagnosis of Mendelian disorders, as it is fast and relatively inexpensive. However, it only investigates the roughly 1–2% of the genome that is translated into protein. Significant progress in the ability to resolve the function of the complete human genome can be expected from several lines of technological development. WGS is being increasingly implemented as the assay of choice for both gene discovery and diagnostic testing. Advantages of WGS include its comprehensiveness, ability to analyse both coding and non-coding sequences that are increasingly understood to have an important role in gene regulation and expression, and promising greater diagnostic yield.^7^ WGS also allows future reanalysis of patients with a negative genomic diagnosis as regions of the genome that were previously unresolved are revealed.^8^ Furthermore, the use of whole transcriptome sequencing will facilitate the identification of genes that are expressed irregularly, which could be an indicator of disease. Accordingly, the ability to combine pharmacogenomics with diagnostics through the same WES and WGS test will ultimately lead to much better and individualised patient care, as well as saving crucial time and money.^9^

## Ethical issues

As Lázaro-Muñoz & Lenk pointed out, psychiatric and neurodevelopmental disorders were some of the phenotypes targeted by the eugenics movement.^10^ Therefore, there is some trepidation among targeted communities on how genetic samples collected may be used in the future. As a result, one of the largest genetic studies, the Spectrum 10 K autism project, has had to be paused for further consultation.^11^ The distinction between genetic testing and pharmacogenomics, where the focus of the latter is on drug response determined by a person's genetics, needs to be made clear from the beginning. It is important to be clear about the wider concerns related to the eugenics movement, so necessary safeguarding is in place when conducting research studies. In setting up and designing genomic research in people with intellectual disabilities, co-production at all phases of projects with experts by experience along with rigorous patient and public involvement are essential to tackle these ethical issues.^12^

## Conclusions

Despite very limited data on effectiveness of pharmacogenomics in people with intellectual disabilities, the increasing evidence base and focus on pharmacogenomics in general suggests that the future looks promising.^13^ In due course it is likely to add another layer of safety in the prescribing of psychotropic medications for this population. This may take a precedence as people with intellectual disabilities are more likely to be on psychotropic medications over the long term compared with their peers without intellectual disabilities. A wider discussion involving all stakeholders, including patients and carers, on the use of pharmacogenetic testing and evaluation of its effectiveness in making tangible differences to prescribing practices are important steps forward. This, along with careful consideration of most the appropriate medication in addition to other factors considered in day-to-day prescribing, may reduce the risk of side-effects, which people with intellectual disabilities often find hard to communicate. This may be another step towards personalisation of medicine for people with intellectual disabilities.

## Data Availability

Data availability is not applicable to this article as no new data were created or analysed in this study.
